# Mesenchymal Stromal Cells in Immunometabolic Regulation: A Review of Itaconate-Mediated Mechanisms

**DOI:** 10.1007/s12015-026-11142-4

**Published:** 2026-05-01

**Authors:** Rosana Lopes Rodrigues Amon, Giulia Toscano Giannini, Sumara de Freitas, Ricardo Ambrósio Fock

**Affiliations:** Department of Clinical and Toxicological Analysis, Faculty of Pharmaceutical Sciences, University of São Paulo, Avenida Lineu Prestes, 580 - Bloco 17, São Paulo, SP 05508-900 Brazil

**Keywords:** Itaconate, Immunometabolism, Mesenchymal stromal cells, Macrophage, Inflammation

## Abstract

Immunometabolism has emerged as a central regulator of immune responses, linking cellular metabolism to inflammatory signaling and tissue homeostasis. Among tricarboxylic acid (TCA) cycle–derived metabolites, itaconate has gained recognition as an important metabolic feedback regulator promoting inflammatory resolution. Mesenchymal stromal/stem cells (MSCs) are multipotent cells widely recognized for their immunomodulatory and regenerative properties, primarily mediated through paracrine signaling and metabolic adaptation. Increasing evidence indicates that MSC immunoregulatory function is closely associated with metabolic reprogramming involving glycolysis, mitochondrial activity, lipid metabolism, and amino acid pathways. Within this context, itaconate has emerged as a potential metabolic interface linking innate immune activation to MSC function. This narrative review summarizes current evidence supporting both direct and indirect interactions between itaconate signaling and MSC biology. Itaconate and its derivatives influence MSC viability, apoptosis resistance, differentiation potential, and redox balance, while indirectly modulating macrophage polarization and inflammatory microenvironment remodeling through extracellular vesicles and paracrine communication. Despite these advances, critical questions remain regarding endogenous itaconate production by MSCs and its effects on MSC secretome composition and immunoregulatory activity. A deeper understanding of the itaconate–MSC axis may enable metabolic preconditioning strategies aimed at enhancing MSC-based therapies for inflammatory and immune-mediated diseases.

## Itaconate as a Central Immunometabolic Regulator

Metabolic reprogramming is a hallmark of innate immune activation and resolution. Rather than serving exclusively as a source of energy, intermediary metabolism actively shapes immune cell signaling and functional outcomes [1]. Among tricarboxylic acid (TCA) cycle–derived metabolites, itaconate has emerged as a key endogenous regulator of inflammation [2].

**Fig. 1:**
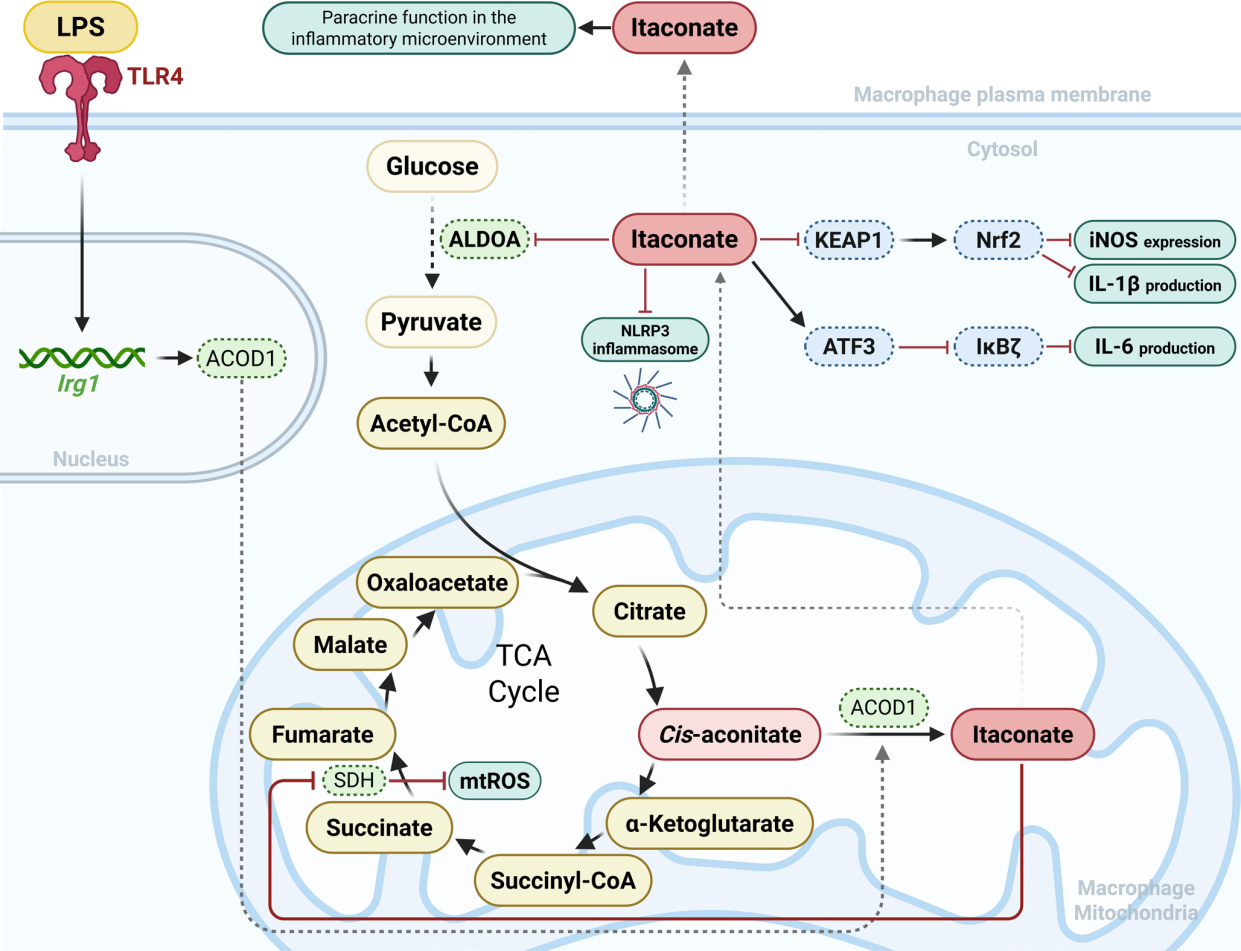
LPS-induced itaconate synthesis and its immunometabolic regulatory role in macrophages. Lipopolysaccharide (LPS) stimulation of macrophages via Toll-like receptor 4 (TLR4) induces *Irg1* expression, encoding aconitate decarboxylase 1 (ACOD1). ACOD1 catalyzes the conversion of cis-aconitate into itaconate in the mitochondria. Mitochondrial itaconate inhibits succinate dehydrogenase (SDH, Complex II), reducing succinate oxidation, mitochondrial ROS production, and downstream inflammatory signaling. Cytosolic itaconate can modulate metabolism by inhibiting aldolase A (ALDOA), decreasing glycolytic flux, and may exert anti-inflammatory effects by modifying KEAP1, stabilizing Nrf2, and suppressing pro-inflammatory mediators such as iNOS and IL-1β. Independently of Nrf2, itaconate induces ATF3, inhibiting IκBζ and reducing IL-6 production. It also inhibits NLRP3 inflammasome activation. Some studies suggest that itaconate or cell-permeable derivatives may influence neighboring cells in a paracrine-like manner

Itaconate is generated from the TCA intermediate cis-aconitate through the enzymatic activity of immune-responsive gene 1 (IRG1), also known as Aconitate Decarboxylase 1 (ACOD1) [2, 3]. Expression of IRG1 is strongly induced in macrophages stimulated by lipopolysaccharide (LPS) and other inflammatory cues, particularly in murine bone marrow–derived macrophages and human monocyte-derived macrophages studied under in vitro conditions [3, 4], leading to intracellular accumulation of itaconate as part of a broader metabolic shift characterized by enhanced glycolysis and mitochondrial remodeling, as shown in Fig. 1. Initially recognized for its antimicrobial activity via inhibition of bacterial isocitrate lyase [5, 6], itaconate is now widely acknowledged as a metabolic mediator that restrains excessive inflammatory responses [4, 7].

One of the best-characterized mechanisms of itaconate is the inhibition of succinate dehydrogenase (SDH) [8, 9], or mitochondrial complex II, which links the TCA cycle to the electron transport chain [10]. By limiting succinate oxidation, itaconate reduces mitochondrial reactive oxygen species (mtROS) production and dampens pro-inflammatory metabolic signaling as demonstrated primarily in LPS-stimulated macrophages in vitro systems and supported by selected in vivo murine models [10, 11]. Beyond enzymatic inhibition, itaconate functions as an electrophilic metabolite capable of modifying cysteine residues in regulatory proteins. Alkylation of Kelch-like ECH-associated protein 1 (KEAP1) promotes the stabilization and activation of the nuclear factor erythroid 2–related factor 2 (Nrf2) transcription factor, leading to antioxidant and cytoprotective gene expression. Nrf2 activation suppresses inflammatory mediators such as Interleukin-1 beta (IL-1β), Hypoxia-inducible factor 1-alpha (HIF-1α), and Nitric oxide synthase 2 (NOS2) [12–14].

Additionally, itaconate modulates inflammatory signaling independently of Nrf2 via the Activating transcription factor 3/ Inhibitor of nuclear factor kappa-B zeta (ATF3/IκBζ) axis, reducing Interleukin-6 (IL-6) production. It also inhibits the NLR family pyrin domain-containing 3 (NLRP3) inflammasome, thereby preventing activation of caspase-1, an evolutionarily conserved protease that cleaves precursor proteins such as interleukin-1β (IL-1β), interleukin-18 (IL-18), and the pyroptosis effector Gasdermin D into their active forms [4, 6, 7]. Caspase-1 plays a central role in innate immunity by initiating inflammatory responses, while Gasdermin D, a pore-forming protein, is crucial for modulating inflammation and pyroptotic cell death [11, 14, 15]. In this way, itaconate suppresses NLRP3 inflammasome activation and modulates inflammatory transcriptional programs through both Nrf2-dependent and independent mechanisms [16] (Fig. 1).

Moreover, itaconate regulates aerobic glycolysis by reducing the activity of aldolase A (ALDOA), leading to decreased glucose consumption and lactate production. In addition, it acts as an inhibitor of fructose-6-phosphate 2-kinase, resulting in glycolytic inhibition in LPS-activated cells, thereby attenuating the inflammatory response through a significant reduction in IL-1β production and inducible nitric oxide synthase (iNOS) expression [2, 11, 17] (Fig. 1). Through these coordinated pathways, itaconate operates as a metabolic negative feedback regulator that promotes immune homeostasis [17, 18].

Importantly, itaconate can be secreted into the extracellular environment, suggesting a paracrine role in shaping the inflammatory microenvironment and influencing neighboring cells [2, 16, 19]. Through these coordinated metabolic and signaling mechanisms, itaconate functions as a key endogenous regulator that restrains inflammation and promotes its resolution [2, 19].

## Mesenchymal Stromal Cells and Immunomodulatory Plasticity

Mesenchymal stromal/stem cells (MSCs) are multipotent progenitor cells identified in bone marrow and subsequently in multiple tissues, including adipose tissue, umbilical cord, placenta, and dental pulp [20–22]. Although originally defined by their differentiation potential into osteogenic, adipogenic, and chondrogenic lineages, MSCs are now primarily recognized for their immunomodulatory properties [23].

The therapeutic effects of MSCs are largely mediated by paracrine and immunoregulatory mechanisms rather than direct tissue replacement. MSCs secrete cytokines, chemokines, growth factors, lipid mediators, and extracellular vesicles that regulate immune cell activation, proliferation, differentiation, and survival [23–25]. Through these mechanisms, MSCs influence both innate and adaptive immune responses across inflammatory and degenerative conditions [26, 27].

Importantly, MSC immunomodulatory activity is not constitutive but highly dependent on environmental cues, a phenomenon commonly described as licensing or functional polarization [26, 27]. Exposure to pro-inflammatory cytokines such as interferon-gamma (IFN-γ) and tumor necrosis factor-alpha (TNF-α) induces a strongly immunosuppressive phenotype characterized by production of mediators including indoleamine-2,3-dioxygenase (IDO), prostaglandin E2 (PGE_2_), nitric oxide (NO) in murine models, interleukin-10 (IL-10), and hepatocyte growth factor (HGF) [28–30]. Under these conditions, MSCs suppress T cell proliferation, inhibit T helper 1 cell (Th1) and T helper 17 cell (Th17) responses, promote regulatory T cell (Treg) expansion, impair dendritic cell maturation, and drive macrophage polarization toward an anti-inflammatory phenotype [31, 32]. Conversely, stimulation with microbial products such as LPS or peptidoglycan (PGN) may transiently induce a pro-inflammatory secretory profile, highlighting the dynamic and context-dependent nature of MSC immunobiology [33, 34]. These observations derive from both murine and human MSCs studied predominantly under in vitro conditions, with partially overlapping but not identical responses across species [28, 31].

Emerging evidence suggests that the functional states of MSCs are tightly linked to their metabolic profile. Glucose metabolism, adenosine triphosphate (ATP) production, lipid metabolism, and amino acid pathways have been shown to directly regulate MSC function and therapeutic potential. Moreover, MSC-mediated immunoregulation is influenced by cellular metabolism, which not only provides energy and biosynthetic precursors but also modulates key signaling pathways. Environmental cues can induce metabolic reprogramming, thereby shaping MSC immunomodulatory capacity and tissue repair functions in inflammatory and immune-mediated diseases [35, 36].

Signaling molecules, inflammatory cytokines, and environmental cues can trigger a metabolic shift in MSCs from oxidative phosphorylation to glycolysis, enhancing their immunoregulatory capacity [36, 37]. In this context, metabolic reprogramming occurs within MSCs themselves and directly supports their ability to produce immunomodulatory mediators [36, 37]. This glycolytic upregulation supports MSC immunomodulatory and regenerative functions, including increased secretion of trophic and immunoregulatory factors, although the magnitude and persistence of this metabolic shift depend on species origin, tissue source, and experimental conditions [35–37]. The serine-threonine kinase mTOR, an evolutionarily conserved sensor of oxygen, nutrients, and growth factors, has emerged as a key regulator of this process [38]. Activation of the mTOR pathway promotes the switch from oxidative phosphorylation to glycolysis, thereby enhancing MSCs immunosuppressive potential [38].

In parallel, mitochondrial activity and ATP metabolism shape MSC-mediated immune responses through tightly interconnected mechanisms [36, 37]. The regulation of redox homeostasis and bioenergetics within MSCs directly determines their immunomodulatory capacity by controlling the production of regulatory mediators and preserving cellular fitness. At the same time, MSCs influence the redox balance and metabolic state of immune cells via paracrine signaling and cell–cell interactions, thereby extending their regulatory effects to the surrounding microenvironment [36, 37]. Thus, while glycolysis sustains proliferation and differentiation capacity, it is also essential for maintaining MSC immunoregulatory functions [36, 37, 39].

Furthermore, alterations in lipid metabolism, particularly changes in phospholipid composition, as well as modulation of amino acid metabolic pathways, including arginine, glutamine, and tryptophan–kynurenine metabolism, which alter T cell activation and differentiation, contribute to the regulation of immune cell function [39, 40]. These effects occur both indirectly, through intrinsic metabolic reprogramming in MSCs that shapes their secretory profile, and directly, via MSC-derived factors that influence immune cell metabolism [39, 40].

Within this immunometabolic framework, itaconate has emerged as a central regulatory metabolite; however, the capacity of MSCs to synthesize it endogenously under inflammatory conditions remains inconclusive, given the low or absent expression of ACOD1/IRG1, the enzyme responsible for its production [4, 41].

In macrophages, ACOD1 expression is strongly induced by inflammatory stimuli such as LPS or TNF-α [4]. Consequently, under these conditions, MSCs not only increase their responsiveness to itaconate signaling but also enhance their capacity to modulate its production within the microenvironment through paracrine interactions [22, 41]. By reconfiguring their bioenergetic state, MSCs integrate itaconate signaling into a broader immunosuppressive program. Thus, this metabolic plasticity plays a pivotal role in regulating immune responses under inflammatory conditions, establishing itaconate as a key mediator within this immunometabolic framework [27, 37, 41].

## Convergence of Itaconate Signaling and MSCs Metabolism

A key aspect of MSC-mediated immunoregulation is the polarization of macrophages. Factors secreted by MSCs promote the shift toward the anti-inflammatory M2 phenotype [24, 25]. Itaconate, generated by macrophages via ACOD1 in response to pro-inflammatory stimuli, regulates inflammation by inhibiting succinate dehydrogenase, activating Nrf2, and reducing reactive oxygen species (ROS) [4, 9]. This metabolite drives macrophages toward a regulatory state, curbing pro-inflammatory cytokine production, supporting tissue repair, and affecting neighboring cells such as MSCs [26, 27]. Because itaconate production is closely tied to macrophage activation and metabolic reprogramming, MSC-induced modulation of macrophage polarization may indirectly alter itaconate levels and signaling within the microenvironment [41–43].

Given that both macrophages and MSCs undergo metabolic remodeling in inflammatory environments, itaconate represents a potential metabolic node linking innate immune activation and MSC function, although current evidence is largely derived from preclinical studies and may not fully recapitulate human pathophysiology. A comprehensive overview of the experimental models and biological systems supporting these findings is provided in Table 1.

**Table 1:** Comprehensive overview of experimental strategies and model systems used to investigate the effects of itaconate signaling on mesenchymal stromal cell (MSC) and immune cell biology

Ref	Author/Year	Study Type	Cell Type/Animal	Species	Model
[3]	Michelucci, A, et al., 2013	Experimental	Primary human monocytic CD14 + cells; Murine microglial BV-2 / SJL Mice	Murine; human	In vitro + In vivo
[4]	Lampropoulou et al., 2016	Experimental	Macrophages (BMDMs) / Mice C57BL/6 N	Murine	Ex vivo
[10]	Paulenda et al., 2025	Experimental	Macrophages (BMDMs); Immortalized BMDMs (iBMDMs); HeLa Kyoto cells / C57BL6/N mice	Murine; human	In vitro + Ex vivo
[11]	Bambouskova et al., 2021	Experimental	Macrophages (BMDMs); Human monocyte-derived macrophages (MoDMs) / C56BL/6 N mice	Murine; human	In vitro + Ex vivo
[24]	Liu et al., 2022	Experimental	Human umbilical cords-MSC; Macrophages by mice bone marrow / C57BL/6J mice	Murine; human	In vitro + Ex vivo
[25]	Liu et al., 2022	Experimental	Human umbilical cords-MSC / C57BL/6J mice	Murine; human	In vivo
[28]	Abbasi-Kenarsari et al., 2020	Experimental	Murine adipose-derived MSCs/ C57BL/6 mice	Murine	In vitro + Ex vivo
[30]	Shahir et al., 2020	Experimental	Macrophages (BMDMs); Lymphocyte; Murine adipose-derived MSCs / C57BL/6 mice; BALB/C mice	Murine	In vitro + In vivo + Ex vivo
[31]	English et al., 2009	Experimental	Human MSC-bone marrow; Human CD4 + T cells	Human	In vitro
[32]	Najar et al., 2010	Experimental	Human MSC-bone marrow; Human mononuclear cells	Human	In vitro
[33]	Kirshenbaum et al., 2008	Experimental	Human mononuclear cells CD34+; Mouse bone marrow-derived mast cells (BMMC) / C57BL/6 mice	Murine; human	In vitro + Ex vivo
[34]	Wang et al., 2024	Experimental	Human placenta-derived mesenchymal stem cells (hP-MSCs) / C57BL/6 mice	Murine; human	In vitro + In vivo
[41]	Wen & Liang, 2024	Experimental	Mouse pulmonary microvascular endothelial cells (PMVECs; CP-M001); Mouse alveolar macrophages; MSCs were purchased from Gibco / C57BL/6 mice	Murine	In vivo + In vitro
[42]	Runtsch et al., 2022	Experimental	Murine bone marrow-derived macrophage (BMDM); Murine resting T cells / C57BL/6J mice	Murine	In vitro + In vivo
[43]	Tabandeh et al., 2022	Experimental	MSC (ADMSC) / Sprague Dawley rats	Murine	In vitro + Ex vivo
[44]	Mills et al., 2018	Experimental	Human PBMCs (macrophages); Bone marrow cells of mice	Murine	In vitro + Ex vivo
[50]	Marchese et al., 2020	Experimental	Primary human MSCs	Human	In vitro
[53]	Li et al., 2025	Experimental	MSC (BMSC) / C57BL/6 mice	Murine	In vitro

Experimental evidence indicates that, at the intracellular level, itaconate and its derivatives directly influence MSC metabolism by enhancing antioxidant responses, stabilizing mitochondrial function, and limiting oxidative stress [43, 44]. Mechanistically, itaconate acts as an anti-inflammatory metabolite that activates the Nrf2 pathway through alkylation of KEAP1, thereby promoting cytoprotective responses [43].

In adipose tissue-derived MSCs, treatment with dimethyl itaconate (DMI) has been shown to increase cell proliferation and modulate the expression of genes associated with redox balance and cellular stress responses. This metabolic and redox reprogramming is critical for preserving MSC functional integrity under inflammatory or ex vivo stress conditions [44]. Consistently, activation of Nrf2-dependent pathways and modulation of redox-sensitive signaling cascades further support the maintenance of MSC viability and immunoregulatory function under these conditions [43, 44].

At the intercellular level, itaconate-mediated reprogramming of macrophages toward a regulatory phenotype may indirectly reinforce MSC-driven immunosuppressive networks [45]. Because MSCs and macrophages engage in bidirectional crosstalk within inflammatory niches, modulation of macrophage metabolism by itaconate may synergize with MSC-derived signals to promote resolution of inflammation [4, 22, 45].

At the tissue level, both MSCs and itaconate contribute to shaping the inflammatory microenvironment through paracrine communication, cytokine modulation, redox signaling, and extracellular vesicle exchange [42, 46]. These convergent pathways support a conceptual framework in which MSCs and metabolic mediators cooperate within an integrated immunometabolic circuit to regulate immune homeostasis [42, 46]. As detailed in the following sections, this autoregulatory process involves bidirectional crosstalk through which MSCs may contribute to the induction of itaconate production in the niche, thereby enhancing their own metabolic capacity.

## Itaconate at the Interface of MSCs Function and Macrophage Immunometabolism

Although most mechanistic insights into itaconate derive from macrophage biology, accumulating evidence indicates that itaconate and its derivatives also directly influence MSC behavior and function, largely derived from in vitro studies using human or murine MSCs and pharmacological derivatives such as dimethyl itaconate (DMI) or 4-octyl itaconate (4-OI) [43, 44].

Importantly, as discussed above, MSCs are not considered major sources of itaconate, as ACOD1/IRG1 expression remains low or absent under both basal and inflammatory conditions [4, 41]. Instead, MSCs regulate itaconate availability within the microenvironment primarily through their interactions with immune cells, particularly macrophages. Through the release of soluble factors and extracellular vesicles, MSCs promote IRG1/ACOD1 expression in macrophages, thereby enhancing endogenous itaconate production [3, 16, 41]. These signals also drive macrophage polarization and metabolic reprogramming, reinforcing an anti-inflammatory phenotype. Concurrently, MSCs are highly responsive to exogenous itaconate and its derivatives, which modulate their proliferation, survival, differentiation, and immunoregulatory functions [30, 41, 43].

This MSC–macrophage crosstalk represents a central axis in immunometabolic regulation. MSC-derived exosomes actively modulate macrophage function and phenotype [2, 10]. Several studies have demonstrated that extracellular vesicles produced by MSCs contain a diverse range of bioactive molecules, including microRNAs, proteins, and metabolites. These vesicles modulate intracellular signaling pathways and, in certain contexts, drive metabolic reprogramming of recipient immune cells, thereby linking MSC-derived extracellular vesicles to immunometabolic regulation and facilitating intercellular communication within the inflammatory microenvironment [21, 30, 41].

Concomitantly, the accumulation of itaconate in macrophages exerts potent regulatory effects, including suppression of NLRP3 inflammasome activation, reduction of mitochondrial reactive oxygen species (ROS) production, and inhibition of NF-κB signaling [2, 28, 30, 41]. In the context of MSC–immune cell interactions, these effects are particularly relevant, as MSCs promote macrophage polarization toward an M2 phenotype and associated metabolic reprogramming, which may include increased IRG1 expression and itaconate production [30, 36, 41, 43].

Rather than directly inducing these anti-inflammatory pathways, MSCs likely act upstream by shaping macrophage activation states that favor itaconate accumulation. The resulting increase in macrophage-derived itaconate contributes to suppression of inflammasome activation and attenuation of excessive inflammatory responses, including cytokine release. Consequently, MSCs exert their therapeutic effects, at least in part, by promoting itaconate production in macrophages rather than producing itaconate themselves [41, 43]. Collectively, these coordinated mechanisms position MSCs as upstream regulators of macrophage metabolism, facilitating the establishment of an anti-inflammatory microenvironment through induction of itaconate synthesis, independently of LPS stimulation [41, 43].

**Fig. 2:**
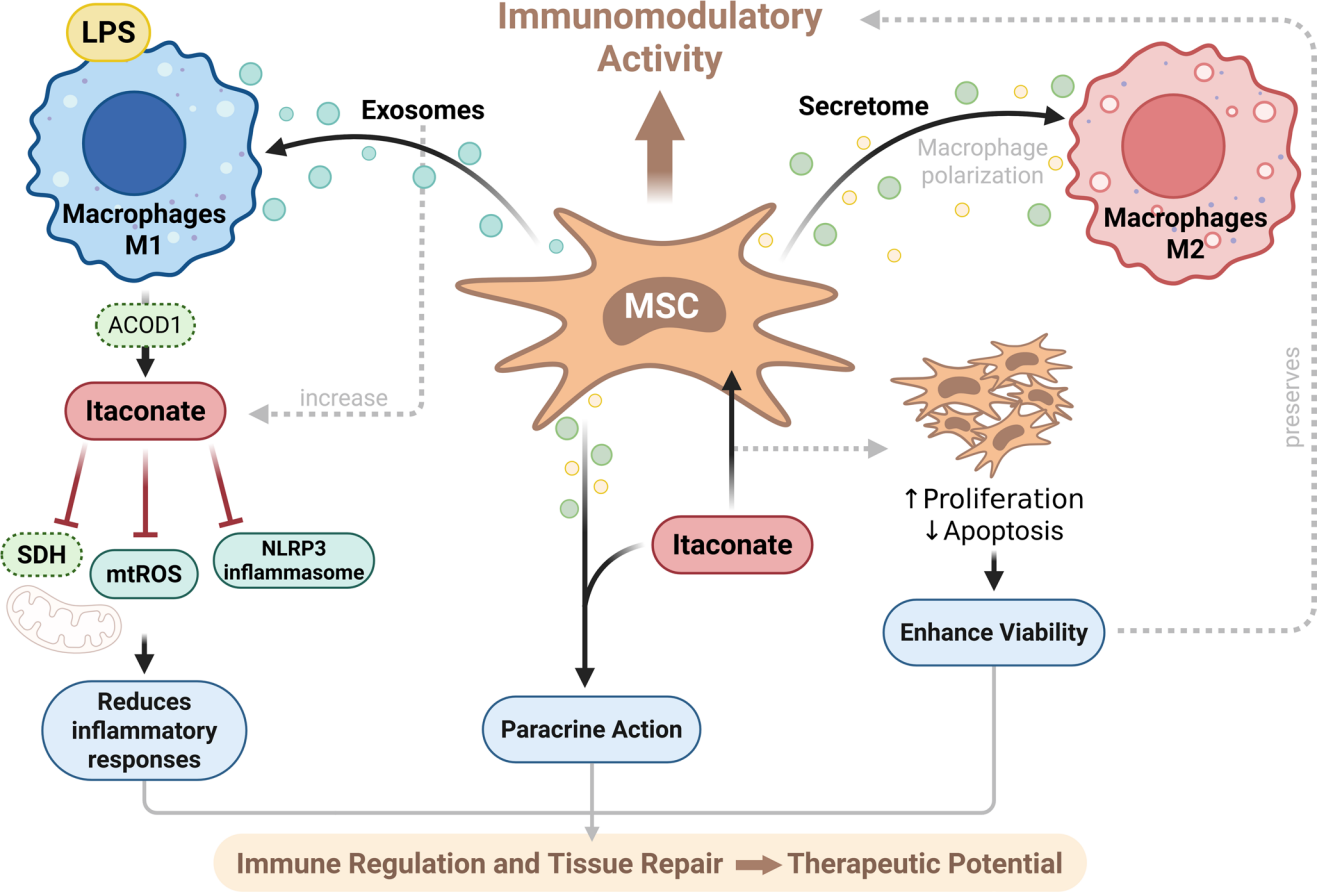
Direct and indirect mechanisms of itaconate in mesenchymal stromal cell–mediated immunomodulation. This schematic illustrates the interplay between itaconate, macrophages, and mesenchymal stromal cells (MSCs) in regulating inflammation and tissue repair. Following LPS stimulation, macrophages polarize toward the M1 pro-inflammatory phenotype, inducing endogenous itaconate synthesis via ACOD1. Itaconate acts autocrinely to attenuate inflammation by inhibiting SDH, reducing mtROS, and suppressing NLRP3 inflammasome activation. While endogenous itaconate has limited membrane permeability, itaconate derivatives such as dimethyl itaconate (DMI) and 4-octyl itaconate (4-OI) can directly enhance MSCs viability by promoting proliferation and reducing apoptosis, supporting their immunomodulatory capacity. MSCs release extracellular vesicles and exosomes containing bioactive molecules that modulate macrophage signaling, potentially contributing to M2 polarization and resolution of inflammation. Together, MSCs-derived factors and macrophage-derived itaconate (or derivatives) act in a paracrine manner to coordinate immune regulation and promote tissue repair, highlighting the therapeutic potential of the MSCs–itaconate axis

Importantly, the integration of MSCs-derived signals with itaconate-driven metabolic pathways suggests a synergistic interaction between stromal and immune compartments [43]. This convergence reinforces anti-inflammatory networks and promotes immune resolution, as illustrated in Fig. 2. Given that macrophage polarization states directly influence tissue repair and inflammatory outcomes, it is plausible that MSC-induced changes in macrophage phenotype modulate endogenous itaconate production, thereby establishing a feedback loop that stabilizes the regulatory microenvironment. This reciprocal interaction underscores the complexity of the immunometabolic landscape in which stromal and immune cells coexist.

In parallel with these indirect effects, itaconate and its derivatives exert direct actions on MSCs, influencing key aspects of their biology supporting survival and functional stability. Tabandeh et al. [43] demonstrated that dimethyl itaconate (DMI) enhances proliferation of adipose-derived MSCs (ADMSCs) and upregulates anti-apoptotic and autophagy-related genes, including *Lc3b*, *Beclin*, and *P62*, while activating *NRF2* signaling. This metabolic reinforcement protects MSCs against oxidative stress and apoptotic death, preserving immunometabolic fitness and enabling continuous production of immunoregulatory mediators during inflammatory challenges.

Given that inflammatory microenvironments impose significant stress on MSC viability, mechanisms that preserve proliferative capacity and confer resistance to apoptosis are essential to maintain their immunomodulatory function and support the resolution of inflammation, enabling continuous production of regulatory mediators and stable licensing under inflammatory cues [27, 47, 48]. Therefore, metabolic reinforcement through itaconate signaling may indirectly enhance the therapeutic stability of MSC preparations [22, 49].

Furthermore, available data indicate that itaconate and its derivatives exert direct metabolic effects on MSCs, influencing their osteogenic and chondrogenic differentiation potential [50]. The preservation of proliferative capacity and protection against apoptosis are essential; however, differentiation capacity represents an additional dimension of MSC-mediated immunoregulation, as it contributes to tissue remodeling and inflammatory control [51, 52].

Evidence from a study by Li et al. [53] in bone marrow-derived MSCs supports this concept. They demonstrated that treatment with 4-octyl itaconate (4-OI) attenuates erastin-induced ferroptosis by restoring the expression of genes involved in lipid detoxification, iron metabolism, and ferroptosis control, including GPX4, FTH1, and SLC7A11. In parallel, 4-OI reduces reactive oxygen species levels, mitigates iron overload, and attenuates mitochondrial damage.

Importantly, beyond modulating ferroptosis-related metabolism, 4-OI restores osteogenic differentiation capacity, which is typically impaired during active ferroptosis, with inhibition of ferroptosis being directly associated with improved differentiation into osteoblasts. These findings indicate that itaconate derivatives preserve MSC viability and metabolic integrity under conditions of oxidative and inflammatory stress [53]. In the context of MSC–immune cell crosstalk, this preservation is particularly relevant, as maintenance of MSC viability and functional stability is essential for sustaining their immunomodulatory capacity, including the continuous secretion of paracrine factors and extracellular vesicles that regulate immune cell behavior [21, 22, 26]. Thus, by limiting ferroptosis, itaconate may indirectly support MSC-driven regulation of immune responses, particularly within inflammatory microenvironments where oxidative stress can compromise stromal cell function [53].

Collectively, these findings position itaconate as a central immunometabolic mediator linking MSCs function, macrophage activation, and microenvironmental regulation. Through indirect modulation of macrophage metabolism and direct effects on MSCs survival, differentiation, and stress responses, itaconate is increasingly recognized as a contributor to the establishment of a regulatory niche that supports tissue repair and immune homeostasis [17, 41, 43, 45]. This bidirectional interaction also suggests the existence of a positive feedback loop, in which MSCs-driven macrophage polarization enhances itaconate production, which in turn stabilizes anti-inflammatory networks and sustains MSC therapeutic efficacy. Nevertheless, it is important to acknowledge that much of the current understanding derives from heterogeneous preclinical experimental systems, encompassing differences in species, cell sources, and in vitro versus in vivo models, which should be carefully considered when interpreting the translational relevance of these findings.

## Perspectives and Future Directions

Although the available data provide a scientific foundation and important insights into the role of itaconate in MSC biology, several critical limitations must be acknowledged. Most of the methodological evidence addressing MSCs, immune cells, and their interactions with itaconate derives from in vitro studies using murine models. This is particularly relevant in the context of immunometabolism, as metabolic pathways are highly sensitive to species-specific differences, tissue origin, and experimental conditions.

Despite significant advances, important mechanistic questions remain unresolved. It is still unclear whether MSCs express ACOD1 and generate endogenous itaconate under inflammatory licensing conditions, or whether the biological effects observed to date are primarily attributable to exogenously administered derivatives. Furthermore, the impact of itaconate on MSCs secretome composition, including the modulation of IDO activity, PGE_2_ production, cytokine release, and extracellular vesicle cargo, remains insufficiently characterized and warrants systematic investigation.

A major experimental limitation arises from the high polarity of itaconate, which restricts passive membrane diffusion and requires active transport into the cytoplasm to exert its anti-inflammatory effects [9]. To overcome this constraint, cell-permeable derivatives such as dimethyl itaconate (DI), 4-octyl itaconate (4-OI), and 4-monoethyl itaconate (4-EI) have been developed and widely employed in experimental models [18, 41, 54]. However, the use of these derivatives further complicates the interpretation of MSC-specific responses, as their biological effects may not fully recapitulate endogenous itaconate signaling.

In this context, several experimental strategies can be proposed to further explore the MSC–itaconate–immunomodulation axis. These include treating MSCs with itaconate or its derivatives, followed by proteomic and metabolomic analyses of their secretome to define immunomodulatory profiles. In addition, co-culture models combining itaconate-treated MSCs with macrophages could be used to assess changes in macrophage inflammatory signaling and soluble mediator production. Additionally, gene-editing approaches, such as CRISPR/Cas9-mediated silencing of ACOD1 in macrophages, may help determine whether the therapeutic effects of MSCs depend on macrophage-derived itaconate within inflammatory microenvironments.

Importantly, in vivo models will be essential to validate these findings in a physiological context. For instance, the therapeutic efficacy of itaconate-treated MSCs could be evaluated in murine models of inflammatory diseases, such as sepsis, colitis, or bone marrow injury. Additionally, the use of ACOD1-deficient mice or macrophage-specific ACOD1 knockout models may help clarify the contribution of endogenous itaconate to MSC-mediated immunomodulation. Combining these approaches with in vivo metabolic tracing and single-cell transcriptomic analyses could further elucidate the cellular and molecular mechanisms underlying this axis.

Consequently, although currently available studies provide valuable insights, the immunomodulatory effects of itaconate-stimulated MSCs have largely been inferred from indirect parameters, primarily cellular proliferation and differentiation [42]. Direct evaluation of immunomodulatory soluble factor release and secretome remodeling under itaconate stimulation remains limited. Addressing these gaps is essential for achieving a more comprehensive understanding of how itaconate influences MSC immunoregulatory function and therapeutic behavior.

A deeper understanding of the itaconate–MSC interface may ultimately enable metabolic preconditioning strategies aimed at enhancing MSC therapeutic efficacy. By stabilizing redox balance, preserving mitochondrial integrity, and sustaining differentiation potential, itaconate signaling may represent a promising metabolic approach for optimizing cell-based therapies in inflammatory and immune-mediated diseases.

## Data Availability

No datasets were generated or analysed during the current study.
